# Maternal oxidative stress during pregnancy associated with emotional and behavioural problems in early childhood: implications for foetal programming

**DOI:** 10.1038/s41380-023-02284-9

**Published:** 2023-10-16

**Authors:** Cindy Pham, Sarah Thomson, Sung-Tong Chin, Peter Vuillermin, Martin O’Hely, David Burgner, Samuel Tanner, Richard Saffery, Toby Mansell, Sze Bong, Elaine Holmes, Peter D. Sly, Nicola Gray, Anne-Louise Ponsonby, Barwon Infant Study Investigator Group, John Carlin, Mimi Tang, Fiona Collier, Amy Loughman, Sarath Ranganathan, Lawrence Gray

**Affiliations:** 1 Murdoch Children’s Research Institute, Royal Children’s Hospital, University of Melbourne, Parkville, VIC 3052 Australia; 2 Florey Institute, University of Melbourne, Parkville, VIC 3052 Australia; 3 Melbourne School of Population and Global Health, University of Melbourne, Parkville, VIC 3052 Australia; 4 Australian National Phenome Centre, Health Futures Institute, Harry Perkins Institute, Murdoch University, Perth, WA 6150 Australia; 5 School of Medicine, Deakin University, Geelong, VIC 3220 Australia; 6 Barwon Health, Geelong, VIC 3220 Australia; 7 Department of Paediatrics, University of Melbourne, Parkville, VIC 3052 Australia; 8 Child Health Research Centre, University of Queensland, South Brisbane, QLD 4101 Australia

**Keywords:** Molecular biology, Predictive markers, Neuroscience, Psychiatric disorders

## Abstract

Childhood mental disorders, including emotional and behavioural problems (EBP) are increasingly prevalent. Higher maternal oxidative stress (OS) during pregnancy (_mat_OS_preg_) is linked to offspring mental disorders. Environmental factors contribute to _mat_OS_preg_. However, the role of _mat_OS_preg_ in childhood EBP is unclear. We investigated the associations between (i) _mat_OS_preg_ and offspring EBP; (ii) social and prenatal environmental factors and _mat_OS_preg_; and (iii) social and prenatal factors and childhood EBP and evaluated whether _mat_OS_preg_ mediated these associations. Maternal urinary OS biomarkers, 8-hydroxyguanosine (8-OHGua; an oxidative RNA damage marker) and 8-hydroxy-2′-deoxyguanosine (8-OHdG; an oxidative DNA damage marker), at 36 weeks of pregnancy were quantified by liquid chromatography-mass spectrometry in a population-derived birth cohort, Barwon Infant Study (*n* = 1074 mother-infant pairs). Social and prenatal environmental factors were collected by mother-reported questionnaires. Offspring total EBP was measured by Child Behavior Checklist Total Problems T-scores at age two (*n* = 675) and Strengths and Difficulties Questionnaire Total Difficulties score at age four (*n* = 791). Prospective associations were examined by multivariable regression analyses adjusted for covariates. Mediation effects were evaluated using counterfactual-based mediation analysis. Higher maternal urinary 8-OHGua at 36 weeks (_mat_8-OHGua_36w_) was associated with greater offspring total EBP at age four (*β* = 0.38, 95% CI (0.07, 0.69), *P* = 0.02) and age two (*β* = 0.62, 95% CI (−0.06, 1.30), *P* = 0.07). Weaker evidence of association was detected for 8-OHdG. Five early-life factors were associated with both _mat_8-OHGua_36w_ and childhood EBP (*P-*range < 0.001–0.05), including lower maternal education, socioeconomic disadvantage and prenatal tobacco smoking. These risk factor-childhood EBP associations were partly mediated by higher _mat_8-OHGua_36w_ (*P-*range = 0.01–0.05). Higher _mat_OS_preg_, particularly oxidant RNA damage, is associated with later offspring EBP. Effects of some social and prenatal lifestyle factors on childhood EBP were partly mediated by _mat_OS_preg_. Future studies are warranted to further elucidate the role of early-life oxidant damage in childhood EBP.

## Introduction

Childhood mental disorders include mood disorders (i.e., depression and anxiety) and neurodevelopmental disorders such as attention deficit/hyperactivity disorder (ADHD) and autism spectrum disorder (ASD) [1, 2]. Mental disorders are concomitant with emotional and behavioural problems (EBP) [3]. Childhood EBP are classified into two domains: internalising (e.g., depression, anxiety and somatic complaints) and externalising (e.g., inattention, hyperactivity, impulsivity, aggression, opposition and conduct) [4]. The estimated worldwide prevalence of EBP in school-aged children is 20% [5], which is expected to rise as a result of the coronavirus disease 2019 (COVID-19) pandemic [6–8]. During childhood, EBP impacts development, health, quality of life, and education [5]. Longitudinal studies have shown that 25 to 60% of young adults diagnosed with emotional disorders have a history of childhood EBP [9]. EBP during early childhood predicts EBP in adolescence and young adulthood, which are linked to the leading causes of mortality, i.e. substance abuse and suicide [4]. Although EBP can be detected as young as two years of age [10], the majority of the studies in this field have investigated EBP in older children and adolescents. Early prevention, detection, and effective treatment of EBP are key public health priorities.

The *Developmental Origins of Health and Disease* (DOHaD) concept emphasises the role of early-life (i.e., prenatal, perinatal and postnatal) environmental exposures in foetal programming, early brain development, and long-term neuropsychiatric outcomes [11]. Indicators of socioeconomic disadvantage [12] and prenatal factors (e.g., tobacco smoking [13], secondhand smoke (SHS) exposure [14] and prescription medication use [15]) have been shown to contribute to the development of EBP. Similarly, these factors have also been shown to impact downstream biological pathways, including oxidative stress (OS) [16, 17] and inflammation [18]. Many of the relevant prenatal factors are modifiable. Therefore, a deeper understanding of the underlying mechanisms is needed to inform prevention strategies and more effective therapeutics.

OS is an imbalance between the production of free radicals, such as reactive oxygen/nitrogen species (ROS/RNS) and antioxidant capacity that leads to oxidative damage to macromolecules [19]. RNA/DNA is generally repaired, and the oxidised products are excreted in the urine. 8-hydroxy-2′-deoxyguanosine (8-OHdG) is a by-product of oxidative DNA damage [20, 21], and urinary 8-OHdG is an established and widely used biomarker of OS. 8-hydroxyguanine (8-OHGua), a base isomer of 8-OHdG that is reflective of oxidative RNA damage, may be a more suitable biomarker of overall OS due to higher detectable concentrations in biofluids [20, 22].

Previous studies have shown that OS is a key pathway impacted by a range of environmental factors, including low socioeconomic position and prenatal lifestyle factors [17]. OS is also implicated in adverse neurodevelopment [23, 24] and the pathogenesis of EBP, including depression and anxiety [25–27]. OS during pregnancy can impact the mother’s health and compromise foetal development through malnutrition and placental perfusion [28]. Advances in animal studies have demonstrated that maternal OS during pregnancy induces brain and behavioural changes in the offspring in mice [29, 30]. Consistent with this, a recent birth cohort study made up of 512 mother-infant pairs reported a positive association between maternal urinary OS biomarker, 8-isoprostane-prostaglandin-F2α (8-iso-PGF2α), in late pregnancy and social impairments (a proxy indicator for ASD) in children at age four years [24]. However, another cohort study of 273 mother-infant pairs found no evidence of an association between maternal plasma OS (8-OHdG) in mid-to-late pregnancy and child ASD-related problems at age three years [31]. Given the small sample size of previous cohort studies, this link between maternal OS in pregnancy and later offspring EBP remains unclear. Further, there is a lack of investigation on the role of early-life OS, particularly for 8-OHGua, in foetal programming and childhood mental disorders.

To address this evidence gap and by utilising data from a large-scale birth cohort, this study aimed to investigate the prospective associations between (i) maternal urinary OS biomarkers (8-OHGua and 8-OHdG) in pregnancy and subsequent offspring EBP at ages two and four years; (ii) social and prenatal environmental factors and maternal OS biomarkers during pregnancy; and (iii) social and prenatal environmental factors and childhood EBP at ages two and four years, and assess whether these factors are mediated by higher maternal OS in pregnancy.

## Methods

### Study design and participants

The *Barwon Infant Study* (BIS) is a population-derived birth cohort of 1074 mother-infant pairs (10 sets of twins) recruited between June 2010 and June 2013 using an unselected antenatal sampling frame in the Barwon region of Victoria, Australia [32]. Infant exclusion criteria included (i) delivery before 32 weeks of pregnancy; (ii) diagnosis of a genetic disease or major congenital malformation; and (iii) serious illness in the first few days of life. Questionnaire data and clinical and biospecimen measures were collected at birth, four weeks, three, six, nine, 12 and 18 months, and at two and four years of age. Study ethics approval was granted by the Barwon Health Human Research Ethics Committee (HREC 10/24), and written informed consent was provided by all parents or guardians.

### Urine collection and processing

At the 36 weeks of pregnancy appointment, a mid-stream urine sample was collected from mothers in a biospecimens jar (minimum 10 mL). All urine samples were kept refrigerated at 4 °C and processed within 24 h under aseptic conditions. Aliquots of urine were taken and then stored at –80 °C until the time of analysis. Previous studies have reported that these OS biomarkers in urine have not shown diurnal variation [33, 34]. Consistent with this, time of day of urine collection was not associated with maternal OS biomarkers in the present study. Notably, the times from urine collection to processing and storage were recorded and adjusted prior to analysis.

### Oxidative stress biomarkers

Urine samples were used for liquid chromatography-mass spectrometry (LC-MS/MS) performed by the Australian National Phenome Centre (Perth, WA, Australia). In brief, chromatographic separation was performed using an ExionLC^TM^ system (SCIEX; Framingham, MA, USA), reversed-phase separation was performed using a Kinetex C_8_ 2.6 μm 2.1 × 150 mm column (Phenomenex; Lane Cove West, NSW, Australia) at 40 °C, and the mass spectrometry detection with electrospray ionisation was performed using a QTRAP 6500+ system (SCIEX; Framingham, MA, USA). The lower limit of quantification (LOQ) was 1.5 ng/mL for 8-OHGua and 0.9 ng/mL for 8-OHdG (Cayman Chemical; Ann Arbour, MI, USA). Data acquisition was performed using Analyst®1.7.1 and analysed using SCIEX OS Analytics 1.7.0 software (SCIEX; Framingham, MA, USA). The inter-assay coefficient of variation was low ( < 10%) for the quality control measures (Supplementary Material Table S2). OS levels below the LOQ were imputed as the LOQ divided by the square root of 2 [35]. Prior to analysis, the urinary OS biomarkers measures were pre-processed to correct for the (i) time interval between urine collection, processing and storage by fitting a linear model and retaining the residuals; [36] (ii) batch effect; [37] and (iii) urine dilution using specific gravity [38]. The pre-processing step reduced the number of covariates that were used in subsequent adjusted models [39]. Given the left-skewed distribution, a base-2 log transformation was applied for subsequent analyses. For further details, see Supplementary Material Box S1 and Table S1.

### Emotional-behavioural outcomes

#### Child Behavior Checklist at age two years

The *Child Behavior Checklist* (CBCL) for ages 1.5 to 5 years is a widely used parent-reported questionnaire designed to assess EBP in children [40]. The CBCL consists of 99 items scored on a three-point Likert scale (0, *not true*; 1, *somewhat or sometimes true;* or 2, *very true or often true*) with high test-retest consistency [40, 41]. Parent-reported scores have been found to strongly correlate with direct measures of child behaviour [42]. Evaluation is based on seven syndrome scales, three composite scores and five Diagnostic and Statistical Manual of Mental Disorders, Fifth Edition (DSM-5)-based orientated scales. The current study primarily focuses on the composite Total Problems scale (referred to as total EBP at age two (tEBP_2y_) hereafter) that summarises all syndrome scale scores. Age-normalised T-scores were employed; this is consistent with our previous work [18, 43, 44]. The CBCL was completed for 675 of the 837 children (81%) reviewed at two years.

#### Strengths and Difficulties Questionnaire at age four years

The *Strengths and Difficulties Questionnaire* (SDQ), preschool version, is a widely used parent-reported questionnaire used to assess EBP in children [45]. The SDQ consists of 25 items, scored on a three-point Likert scale (0, *not true*; 1, *somewhat true*; or 2, *certainly true*) with validated psychometric properties [46]. Evaluation is based on five subscales and a Total Difficulties score (referred to as total EBP at age four (tEBP_4y_) hereafter). The SDQ was completed for 791 of the 847 children (93%) reviewed at four years.

#### Endophenotype outcomes

We first examined tEBP_2y_ and tEBP_4y_. We then additionally investigated specific endophenotype outcomes within the broader total EBP phenotype, including CBCL DSM-5 Depressive Problems at age two years and SDQ Emotional Symptoms at age four years. These are important indicators of emotional disorder symptoms in young children [6–8].

### Other factors

Early-life social and environmental factors linked to childhood EBP were selected based on a priori knowledge from previous work in this cohort [18, 43, 44, 47, 48]. Key early-life factors domains in this study included sociodemographic, household composition, and maternal prenatal factors that were measured by a questionnaire at 28–36 weeks of pregnancy [32]. Parental education status was dichotomised as no university (lower education) vs. university degree. Mean household income reported over pregnancy and the first postnatal year was standardised to have a mean of zero and a standard deviation (SD) of one. Residential socioeconomic disadvantage was measured by the Socio-Economic Indexes for Areas Index of Relative Socioeconomic Disadvantage (SEIFA-IRSD) in Australia in 2016; scores in the lowest tertile indicate greater disadvantage [49]. Prenatal maternal perceived stress was measured by the Perceived Stress Score (PSS) during pregnancy [50]. Home environmental toxicant measures, including air freshener, paint and herbicide use, are described elsewhere [47].

### Statistical analysis

Separate multivariable linear regression models were used to assess the associations between (i) maternal urinary OS biomarkers at 36 weeks of pregnancy, 8-OHGua (_mat_8-OHGua_36w_) and 8-OHdG (_mat_8-OHdG_36w_), and continuous EBP outcomes at ages two and four years; and (ii) early-life factors and both maternal OS biomarkers during pregnancy or childhood EBP. Robust regression models were utilised to account for possible heteroscedasticity. In the minimally adjusted model, adjustment factors included the child’s sex and process factors for the EBP outcomes (postnatal age at the time of behavioural assessment) and those for the OS exposures (gestational age at the time of urine collection). These were applied to minimise measurement error, which is in line with our previous work in this cohort [18, 44].

Given the low-knowledge environment with early-life OS, we used data-derived approaches to determine whether factors were possible antecedents, mediators or confounders of the OS-EBP associations, as previously detailed elsewhere [51, 52]. Factors were selected based on a priori knowledge from this cohort [18, 43, 44, 47, 48]. The fully adjusted model also included maternal age and household income. Factors independently associated with maternal OS during pregnancy and also with childhood EBP may potentially exert an effect on childhood EBP through the OS causal pathway, and this was examined using the R statistical package ‘*mediation*’ [53]. Additional analyses were conducted (Supplementary Material Tables S6 and S7). *P*-values were reported as exact values, serving as a continuous measure of the compatibility of a null model with the sample data [54, 55]. Analyses were conducted on Stata 16.1 software (StataCorp, College Station, TX) and R version 4.1.0, 2021 (R Core Team).

## Results

### Participant characteristics

**Fig. 1:**
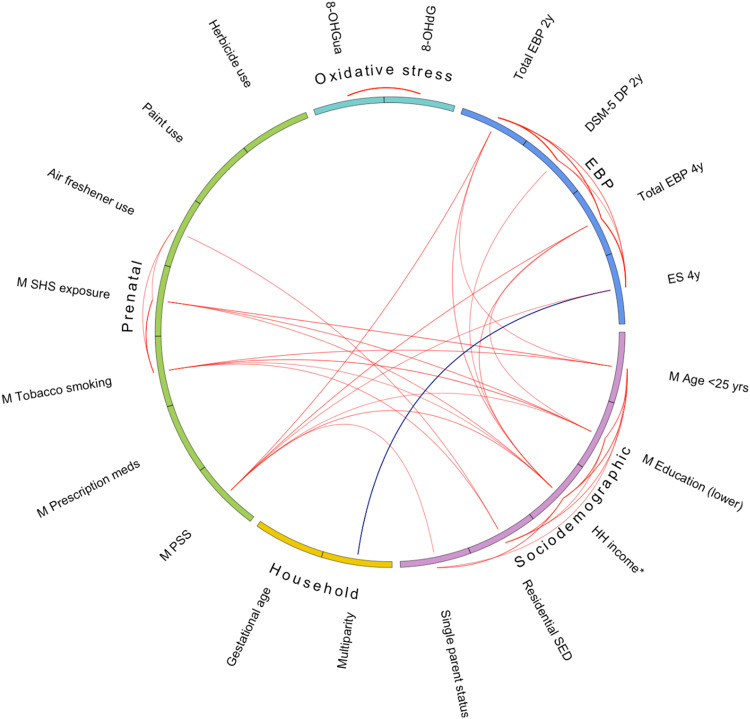
Correlogram of key early-life factors, OS biomarkers at 36 weeks of pregnancy and EBP at ages two and four years. Key early-life factors are represented as sociodemographic, household composition, and prenatal. Maternal OS biomarkers at 36 weeks of pregnancy are indicated as the OS domain. Emotional and behavioural problems are indicated as the EBP domain. Lines outside of the circle indicate intra-domain correlations. Lines inside the circle indicate inter-domain correlations. For both intra- and inter-domains, Pearson’s correlation limits are  ≤ –0.10 and ≥ 0.10. Red lines indicate positive correlations. Blue lines indicate negative correlations. Maternal education level (lower indicates no university degree). 8-OHdG 8-hydroxy-2-deoxyguanosine, 8-OHGua 8-hydroxyguanosine, DP depressive problems, ES emotional symptoms, DSM-5 Diagnostic and Statistical Manual of Mental Disorders Version 5, EBP emotional and behavioural problems, HH household, M maternal, meds medications, PSS Perceived Stress Score, SED socioeconomic disadvantage, SHS secondhand smoke, yrs years.

Key characteristics of the study sample are listed in Table 1, and an extended list of factors is presented in Supplementary Material Table S3; a total of 62 early-life factors were examined. A flowchart of participants in the cohort study is shown in Supplementary Material Fig. S1. Among the mothers of the children with emotional and behavioural assessment measures at two years (*n* = 675) and at four years (*n* = 791), the majority were older than 25 years of age, most had completed a university degree, and most were non-smokers. _Mat_8-OHGua_36w_ had a mean of 2.8 ng/mL and a SD of 1.5, and _mat_8-OHdG_36w_ had a mean of 1.2 ng/mL and a SD of 1.9. Correlations between key early-life factors, log-transformed maternal OS biomarkers during pregnancy and childhood EBP are shown in Fig. 1.

**Table 1:** Participant characteristics.

	2 years (*n* = 675)		4 years (*n* = 791)	
Key early-life factors	*N*	*n* (%) or mean [SD] or GM {GSD}	*N*	*n* (%) or mean [SD] or GM {GSD}
Sociodemographic				
Maternal age (<25 years)	674	32 (4.7)	790	47 (5.9)
Maternal education (lower)^a^	672	265 (39.4)	787	335 (42.6)
Mean household income^b^	669	9.7 [3.2]	787	9.6 [3.3]
Residential SED (lowest tertile)^c^	667	196 (29.4)	783	239 (30.5)
Household composition				
Single-parent family	675	18 (2.7)	791	20 (2.5)
Multiparity	674	372 (55.2)	790	434 (54.9)
Prenatal				
Gestational age at birth, weeks	675	39.5 [1.5]	791	39.5 [1.5]
Perceived stress, score^d^	533	17.7 [6.5]	606	18.1 (6.6)
Prescription medication use	657	330 (50.2)	773	380 (49.2)
Tobacco smoking	673	76 (11.3)	788	97 (12.3)
SHS exposure	662	81 (12.2)	775	109 (14.1)
Air freshener use	487	247 (50.7)	551	276 (50.1)
Paint use	484	143 (29.5)	549	162 (29.5)
Herbicide use (weed killer)	669	108 (16.1)	784	122 (15.6)
Maternal OS biomarkers				
8-OHGua, ng/mL	579	2.8 {1.5}	653	2.8 {1.6}
8-OHdG, ng/mL	558	1.2 {1.9}	622	1.2 {1.9}
Child EBP outcomes				
CBCL T-score at 2 years:				
Total problems	675	43.8 [8.8]		
DSM-5 Depressive problems	675	52.8 [4.0]		
SDQ score at 4 years:				
Total difficulties			791	8.1 [4.6]
Emotional symptoms			791	1.5 [1.6]

*8-OHdG* 8-hydroxy-2-deoxyguanosine, *8-OHGua* 8-hydroxyguanosine, *AUD* Australian dollars, *CBCL* Child Behavior Checklist, *DSM-5* Diagnostic and Statistical Manual of Mental Disorders Version 5, *GM* geometric mean, *EBP* emotional and behavioural problems, *GSD* geometric standard deviation, *ng/mL* per 1 nanogram per millilitre, *N* number of participants with measured data, *OS* oxidative stress, *SD* standard deviation, *SDQ* Strength and Difficulties Questionnaire, *SED* socioeconomic disadvantage, *SHS* secondhand smoke.

^a^Maternal education level (lower indicates no university degree).

^b^Mean household income indicates mean household income during pregnancy and the first year, by $10,000 AUD.

^c^Residential SED (lowest tertile indicates greater disadvantage).

^d^Maternal perceived stress was measured by the Perceived Stress Score.

### Associations between maternal OS biomarkers during pregnancy and childhood EBP

The prospective associations between maternal OS biomarkers during pregnancy and childhood EBP are shown in Table 2. Higher _mat_8-OHGua_36w_ was associated with greater tEBP_4y_ (regression mean change (*β)* = 0.42 points per 1-SD increase in 8-OHGua, 95% CI (0.10, 0.73), *P* = 0.01) in the minimally adjusted model. This association was marginally attenuated in the fully adjusted model. Similar but weaker patterns were found for _mat_8-OHGua_36w_ and tEBP_2y_. Positive associations were also present between _mat_8-OHGua_36w_ and the specific endophenotype outcomes, i.e., DSM-5 Depressive Problems at age two and Emotional Symptoms at age four. Both persisted in the fully adjusted model. Weaker evidence of association was generally found between _mat_8-OHdG_36w_ and EBP in early childhood compared to _mat_8-OHGua_36w_ (*P* > 0.05). Given the stronger estimated effects for _mat_8-OHGua_36w_ compared to _mat_8-OHdG_36w_ (Table 2), we focused therefrom on 8-OHGua as our primary OS biomarker in subsequent analyses.

**Table 2:** The prospective associations between maternal OS biomarkers at 36 weeks and total EBP at ages 2 and 4 years.

	Maternal OS Biomarkers							
	8-OHGua, ng/mL				8-OHdG, ng/mL			
EBP Outcomes	*β* (95% CI)^*^	*P*	*β* (95% CI)^†^	*P*	*β* (95% CI)^*^	*P*	*β* (95% CI)^†^	*P*
CBCL T-score at 2 years								
Total EBP	0.63 (−0.04, 1.31)	0.06	0.62 (−0.06, 1.30)	0.07	0.07 (−0.81, 0.95)	0.88	0.18 (−0.70, 1.06)	0.69
DSM-5 Depressive problems	0.38 (0.10, 0.67)	0.01	0.37 (0.09, 0.65)	0.01	0.34 (−0.07, 0.76)	0.10	0.38 (−0.03, 0.80)	0.07
SDQ score at 4 years								
Total EBP	0.42 (0.10, 0.73)	0.01	0.38 (0.07, 0.69)	0.02	0.08 (−0.35, 0.51)	0.71	0.12 (−0.30, 0.53)	0.59
Emotional symptoms	0.14 (0.02, 0.25)	0.02	0.13 (0.01, 0.24)	0.03	0.09 (−0.06, 0.25)	0.22	0.09 (−0.06, 0.24)	0.23

*8-OHdG* 8-hydroxy-2-deoxyguanosine, *8-OHGua* 8-hydroxyguanosine, *CBCL* Child Behavior Checklist, *CI* confidence interval, *DSM-5* Diagnostic and Statistical Manual of Mental Disorders Version 5, *EBP* emotional and behavioural problems, *ng/mL* 1 nanograms per millilitre, *OS* oxidative stress, *SDQ* Strengths and Difficulties Questionnaire.

*Minimally adjusted for child’s sex, gestational age at urine collection, and child’s age at the time of behavioural assessment.

^†^Fully adjusted additionally for maternal age and household income.

### Early-life factors associated with maternal 8-OHGua during pregnancy and childhood EBP

Key early-life factors that were associated with _mat_8-OHGua_36w_ and childhood EBP are presented in Table 3. Factors are categorised into three domains: sociodemographic, household composition and prenatal. Factors that were independently associated with (i) _mat_8-OHGua_36w_ and (ii) one or both total EBP outcomes included maternal prenatal tobacco smoking, maternal prenatal SHS exposure, lower maternal education, residential socioeconomic disadvantage and prenatal prescription medication use. Factors that were only associated with _mat_8-OHGua_36w_ included gestational age at birth, prenatal paint use and herbicide use, while factors only associated with both EBP outcomes included maternal age, household income, single-parent family, multiparity, maternal prenatal perceived stress and air freshener use. For an extended list of examined early-life factors (Supplementary Material Tables S3 and S4).

**Table 3:** Individual associations between early-life factors and (i) maternal 8-OHGua during pregnancy and (ii) child total EBP in early childhood.

	Maternal OS Biomarker		Child Total EBP			
	8-OHGua, ng/mL		2 years		4 years	
Key early-life factors	*β* (95% CI)^*^	*P*	*β* (95% CI)^†^	*P*	*β* (95% CI)^†^	*P*
Sociodemographic						
Maternal age (<25 years)	0.11 (−0.23, 0.44)	0.53	6.31 (2.87, 9.74)	<0.001	2.27 (0.78, 3.75)	0.003
Maternal education (lower)^a^	0.17 (0.02, 0.32)	0.02	1.46 (0.09, 2.83)	0.04	1.40 (0.74, 2.06)	<0.001
Household income, $10,000 AUD^b^	0.04 (−0.04, 0.11)	0.37	1.84 (1.14, 2.54)	<0.001	0.89 (0.56, 1.22)	<0.001
Residential SED (lowest tertile)^c^	0.22 (0.06, 0.38)	0.01	0.89 (−0.63, 2.42)	0.25	0.80 (0.08, 1.52)	0.03
Household composition						
Single-parent family	−0.37 (−0.92, 0.19)	0.20	3.88 (0.31, 7.46)	0.03	3.81 (1.76, 5.86)	<0.001
Multiparity	−0.06 (−0.21, 0.08)	0.40	−1.48 (−2.81, −0.14)	0.03	−1.28 (−1.91, −0.64)	<0.001
Prenatal						
Gestational age, weeks	0.07 (0.01, 0.14)	0.02	0.01 (−0.46, 0.49)	0.96	−0.05 (−0.28, 0.18)	0.67
Perceived stress, score	−0.001 (−0.01, 0.02)	0.97	0.31 (0.20, 0.41)	<0.001	0.15 (0.10, 0.21)	<0.001
Prescription medication use	0.16 (0.01, 0.30)	0.04	−0.04 (−1.39, 1.32)	0.96	0.85 (0.20, 1.49)	0.01
Tobacco smoking	0.31 (0.09, 0.53)	0.006	3.36 (1.28, 5.43)	0.002	1.39 (0.38, 2.40)	0.007
SHS exposure	0.19 (0.003, 0.38)	0.05	1.95 (−0.08, 3.97)	0.06	1.59 (0.62, 2.56)	0.001
Air freshener use	0.13 (−0.02, 0.29)	0.10	1.94 (0.32, 3.55)	0.02	1.17 (0.41, 1.94)	0.003
Paint use	−0.21 (−0.38, −0.03)	0.02	0.89 (−0.82, 2.6)	0.31	0.38 (−0.47, 1.22)	0.38
Herbicide use (weed killer)	−0.22 (−0.42, −0.01)	0.04	−0.56 (−2.31, 1.19)	0.53	0.01 (−0.89, 0.91)	0.98

For an extension of associations between early-life factors and maternal OS biomarkers and childhood EBP, see Supplementary Materials Table S4.

*8-OHGua* 8-hydroxyguanosine, *AUD* Australian dollars, *CI* confidence interval, *EBP* emotional and behavioural problems, *ng/mL* per 1 nanogram per millilitre, *OS* oxidative stress, *SED* socioeconomic disadvantage, *SHS* secondhand smoke.

*Minimally adjusted for child’s sex and gestational age at the time of urine collection.

^†^Minimally adjusted for child’s sex and time of behavioural assessment.

^a^Maternal education level (lower indicates no university degree).

^b^Household income (by $10,000 AUD) was standardised to have a mean of 0 and SD of 1.

^c^Residential SED (lowest tertile indicates greater disadvantage).

### Maternal 8-OHGua during pregnancy partly mediates the effects of socioeconomic disadvantage and prenatal lifestyle factors on childhood EBP

**Fig. 2:**
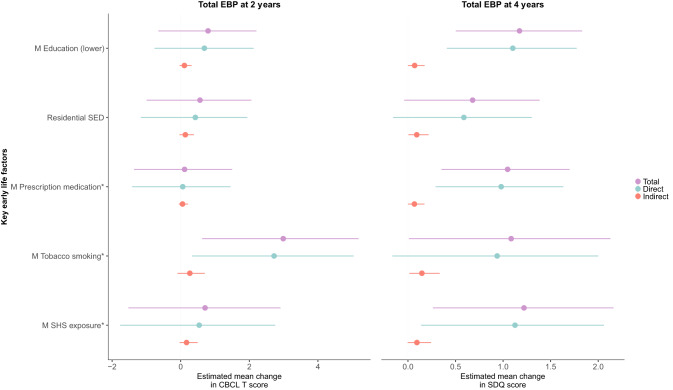
Associations between key early-life factors and total EBP at ages 2 and 4 years, mediated by OS biomarker 8-OHGua at 36 weeks. ^*^Prenatal factors. Maternal education (lower indicates no university degree). Residential SED represents greater disadvantage. Minimally adjusted for child’s sex, gestational age at urine collection, and child’s age at the time of behavioural assessment. 8-OHGua 8-hydroxyguanosine, CBCL Child Behavior Checklist, EBP emotional and behavioural problems, M maternal, OS oxidative stress, SED socioeconomic disadvantage, SDQ Strengths and Difficulties Questionnaire, SHS secondhand smoke.

**Fig. 3:**
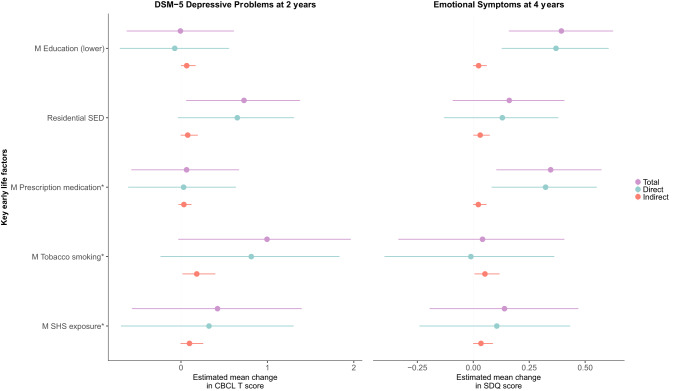
Associations between key early-life factors and emotional problems at ages 2 and 4 years, mediated by OS biomarker 8-OHGua at 36 weeks. ^*^Prenatal factors. Maternal education (lower indicates no university degree). Residential SED represents greater disadvantage. Minimally adjusted for child’s sex, gestational age at urine collection, and child’s age at the time of behavioural assessment. 8-OHGua 8-hydroxyguanosine, CBCL Child Behavior Checklist, DSM-5 Diagnostic and Statistical Manual of Mental Disorders Version 5, M maternal, SED socioeconomic disadvantage, OS oxidative stress, SDQ Strengths and Difficulties Questionnaire, SHS secondhand smoke.

_Mat_8-OHGua_36w_ was examined as a potential mediator of the associations between the key early-life factors and childhood EBP (Figs. 2 and 3). For tEBP_4y_, factors with significant indirect effect (mediation effect) estimates included maternal prenatal tobacco smoking (*β* = 0.15, 95% CI (0.02,0.34), *P* = 0.01), maternal SHS exposure during pregnancy (*β* = 0.09, 95% CI (0.001, 0.24), *P* = 0.05), lower maternal education (*β* = 0.07, 95% CI (0.003, 0.18), *P* = 0.04), greater residential socioeconomic disadvantage (*β* = 0.09 (0.009, 0.21), *P* = 0.02) and prescription medication use (*β* = 0.07, 95% CI (0.002, 0.17), *P* = 0.04). Between 6–12% of the effects of each of these factors were partly mediated by higher _mat_8-OHGua_36w_ (Fig. 2 and Supplementary Material Table S5). These mediation effects were similar for both DSM-5 Depressive Problems at age two and Emotional Symptoms at age four years (Fig. 2). Thus, these findings suggest that the adverse effects of these factors on offspring risk of EBP in early childhood, including emotional symptoms, play a small role in the causal pathway by partly acting through greater maternal oxidative stress during pregnancy. These findings were not materially altered with various additional analyses; see Supplementary Material Tables S6 and S7.

## Discussion

To our knowledge, this is the first study to examine the longitudinal associations between maternal OS biomarkers, 8-OHGua and 8-OHdG, during pregnancy and persistent total EBP in early childhood. Our findings suggest that higher _mat_8-OHGua_36w_, a measure of oxidative RNA damage, was positively associated with children’s total EBP at ages two and four years. _Mat_8-OHGua_36w_ was also associated with greater emotional problems such as depression and anxiety in early childhood. The estimates were altered by less than 10% after adjusting for potential confounders. We also found weak evidence of an association between _mat_8-OHdG_36w_ and childhood EBP, suggesting a limited role for this biomarker in predicting later EBP in the child. We also identified a number of modifiable risk factors for EBP that were partially mediated by higher _mat_8-OHGua_36w_, including socioeconomic disadvantage (i.e., lower maternal education and residential socioeconomic disadvantage) and maternal prenatal lifestyle factors (i.e., tobacco smoking, SHS exposure and prescription medication use). Together, our findings demonstrate that social and prenatal lifestyle environmental factors may play a contributing role in the development of EBP in the offspring and may be influenced by higher maternal oxidative RNA damage during pregnancy.

Few studies have investigated the impact of maternal OS during pregnancy on the offspring’s emotional-behavioural [24] and neurodevelopmental outcomes [31]. A longitudinal study found that increased levels of an OS biomarker (8-iso-PGF2α) and a proxy biomarker for OS, prostaglandin-F2α (PGF2α), measured in the third trimester of pregnancy were associated with greater social impairment, a proxy indicator for ASD symptoms in children at age four years [24]. Notably, the authors adjusted for several early-life factors, including maternal education and tobacco smoking. To examine the influence of lower maternal education further in this study, we conducted a sensitivity analysis that evaluated whether the four risk factors remained mediated through OS after additional adjustment for lower maternal education in this study (Supplementary Material Table S6), considering that lower maternal education may be acting through non-OS pathways [24]. There was evidence that the mediation effects persisted for prenatal tobacco smoking and residential socioeconomic disadvantage, but there was weaker evidence for prenatal prescription medication use and prenatal SHS exposure. A recent birth cohort study reported increases in both the ratio of glutathione (GSH) and glutathione disulfide (GSSG)(GSH:GSSG ratio), reflective of antioxidant balance, and plasma 8-OHdG levels during pregnancy were modestly associated with ASD-related symptoms in children at three years [31]. However, we were not able to replicate this finding in this study at ages two and four. This may reflect the higher sensitivity of plasma 8-OHdG, i.e., reflective of the steady state of DNA damage and is detected at lower levels than in urine, while urinary 8-OHdG levels are more indicative of the total DNA damage [56, 57]. Murine studies have found that increased maternal OS during pregnancy is associated with adverse offspring brain and behavioural development, particularly with emotional disorders [30]. In adults, several meta-analyses have also shown that increased OS is associated with depression [25–27]. This is further supported by our finding that maternal OS biomarkers levels during pregnancy were associated with increased depressive symptoms in early childhood. Collectively, these findings highlight the significant impact of early OS during critical periods of plasticity in the emotional-behavioural and neurodevelopment trajectory of the offspring, particularly linked mood disorders in children.

We found a positive association between oxidative RNA damage biomarker 8-OHGua and EBP in children, but we did not find a similar association with oxidative DNA damage biomarker 8-OHdG. Although urinary 8-OHdG is widely used, urinary 8-OHGua may, in fact, be a better biomarker for oxidative damage [22], as RNA is more vulnerable than DNA to oxidative damage; its structure is mostly single-stranded and less compact, making it more accessible to free radicals [58]. In adults, 8-OHGua levels are several hundred-fold higher than 8-OHdG levels in saliva [20] and given the moderate correlation between the measurement of OS in saliva and in urine [59], this may be a plausible explanation as to why 8-OHGua had a stronger signal than 8-OHdG in this study. In addition, 8-OHdG has been shown to represent whole-body oxidative DNA damage [56]. The majority of the data on 8-OHGua as an OS biomarker relates to adults with neurodegenerative diseases [60] and emotional disorders [61]. Much less is known regarding the importance of 8-OHGua in children [62], and in this context, our findings highlight the value of 8-OHGua as an OS biomarker in paediatric research.

The effects of socioeconomic disadvantage (i.e., lower maternal education and greater residential socioeconomic disadvantage) and maternal prenatal lifestyle factors (i.e., tobacco smoking, SHS exposure and prescription medication use) on early childhood EBP were each partially mediated by higher _m_8-OHGua_36w_ highlighting that OS could be one of the unifying pathways for multiple adverse impacts on foetal brain development. Tobacco smoking [63], non-steroidal anti-inflammatory drugs (NSAIDs), antiretroviral agents, antipsychotics, and analgesics [64] have each been shown to induce OS. While we were able to replicate several of these findings in our cohort of pregnant women, we were unable to detect associations between prenatal antidepressants, antibiotics, or paracetamol exposure and OS in this study (Supplementary Material Table S4). Our findings provide the first insights into the role of OS in the foetal programming of mental disorders. This further supports the DOHaD concept that adverse environmental effects in early life have detrimental consequences on the offspring that may manifest later in life.

### Strengths and limitations

This study is the largest of its kind to directly assess maternal OS measures during pregnancy and subsequent persistent EBP in young children. The rich array of data assembled in BIS enabled the investigation of a diverse range of early-life social and environmental factors and EBP outcomes. Both the CBCL and SDQ instruments are widely used, validated and clinically relevant measures for the assessment of EBP in children [41, 46]. The CBCL DSM-5 scales have been shown to have good predictive validity for neurodevelopmental disorders [65]. Two OS biomarkers that provided insights into different biological mechanisms were measured in parallel. These OS biomarkers were quantified in non-invasive urine rather than blood, which is prone to auto-oxidation after sampling [66]. Another major strength is the high-quality OS measurements; chromatographic methods are considered the ‘gold standard’ with greater specificity for the isomer of interest [67]. Process factors such as batch effects and the time interval between collection, processing to storage were controlled for to minimise measurement error [51]. Given the known variability of urine dilution measures during pregnancy [68], we controlled for two indicators of urine dilution, specific gravity and freezing-point osmolality, and subsequent analyses gave consistent results. Several modern causal inference approaches were employed: counterfactual-based mediation analyses were used to demonstrate that well-known risk factors of EBP were operating partly through maternal OS during pregnancy to influence EBP outcomes in children, and magnitudes of associations, dose-response patterns, and consistency with past work were considered [51]. Inverse probability weighting was applied to evaluate potential selection bias, and the main results were largely consistent [69].

Limitations of this study included a single measurement of maternal OS biomarkers at 36 weeks of pregnancy, reducing the generalisability to other timepoints during pregnancy or postnatally in children. The oxidative DNA/RNA damage biomarkers measured may only reflect one possible subset of the OS pathway; others, such as lipid peroxidation biomarkers (i.e., isoprostanes), may also be important [24]. The kinetic profiles of urinary oxidative damage biomarkers, 8-OHGua and 8-OHdG, are less well-characterised, particularly in pregnancy. However, 8-OHdG has been reported to be relatively stable [70]. Our cohort was not large enough to examine sex-specific effects that may be relevant [4]. Multiple comparison adjustments were not performed given that not all early-life factors were independent and to minimise the risk of false-negative errors [71]. The availability of genetic data was limited to the child’s genotype [72], and therefore, the mother’s genetic susceptibility to OS could not be examined. Given that the known half-life of oxidant damage measures is short [73], maternal OS measures may not be representative of the child’s OS levels and, therefore, warrants future studies to elucidate the role of OS in children and EBP.

## Conclusion

Higher maternal 8-OHGua biomarker levels, a marker of oxidative RNA damage, in pregnancy were associated with increased total EBP, particularly emotional-related problems, during early childhood. The effects of several social and prenatal lifestyle risk factors on childhood EBP were partially mediated by higher _mat_8-OHGua_36w_, indicating that OS may be a common underlying pathway. Our findings highlight the importance of strategies designed to reduce OS during pregnancy with the goal of optimising emotional-behavioural and neurodevelopmental outcomes in children. Further studies are warranted to replicate these findings, extend our understanding of the determinants and consequences of early OS and inform future intervention trials to prevent childhood mental disorders.

### Supplementary information

Supplementary Material
