# Supplementary material for: Maternal oxidative stress during pregnancy associated with emotional and behavioural problems in early childhood: implications for foetal programming
Source: Mol Psychiatry. 2023 Oct 16;28(9):3760–8. doi: 10.1038/s41380-023-02284-9 (PMC10730421; doi:10.1038/s41380-023-02284-9)
Supplement: Supplementary file 1 — Supplementary Material [file 41380_2023_2284_MOESM1_ESM.docx]

**SUPPLEMENTARY MATERIAL**

**Table of Contents**

**Figure S1.** Flowchart for the selection of participants in this study.

**Box S1.** Details on the experimental methods and materials for the urinary OS measurements by liquid chromatography-mass spectrometry (LC-MS/MS).

**Table S1.** The LC-MS/MS parameters used in the experimental methods.

**Table S2.** Inter-batch precision in the measurement of urinary OS biomarkers

**Table S3.** Extended sample characteristics**.**

**Table S4.** Associations between early life factors with (i) maternal OS biomarkers at 36 weeks of pregnancy; and (ii) child total EBP at ages 2 and 4 years.

**Table S5.** Associations between key early life factors and EBP at ages 2 years and 4 years, mediated by maternal 8-OHGua biomarker levels at 36 weeks of pregnancy, adjusted for relevant covariates.

**Table S6.** Associations between key early life factors and EBP at ages 2 years and 4 years, mediated by maternal 8-OHGua biomarker levels at 36 weeks during pregnancy additional adjusted for lower maternal education.

**Table S7.** Linear regression estimates for the relationship between maternal OS biomarkers at 36 weeks of pregnancy and EBP at ages 2 and 4 years in models: (A) and (B) with an interaction term for OS biomarker and child’s sex; (C) with additional adjustment for maternal weight gain during pregnancy; and (D) where OS biomarkers were corrected for urine osmolality instead of specific gravity.

**Table S8.** Associations between individual child OS-related genes and maternal OS biomarkers at 36 weeks of pregnancy.

**
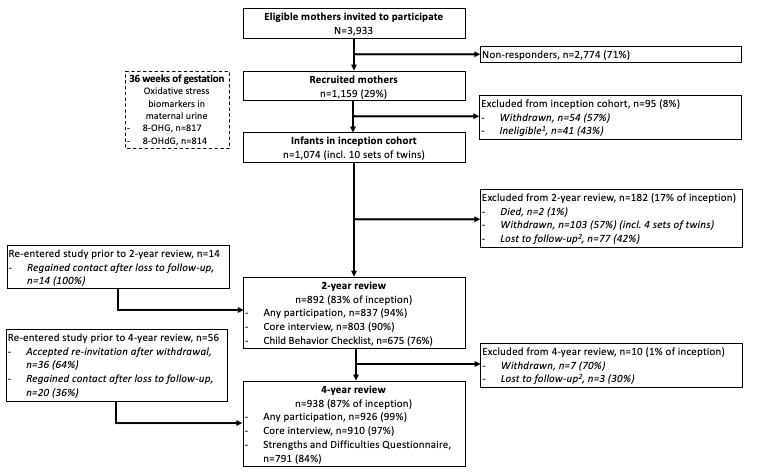
**

**Figure S1.** Flowchart for the selection of participants in this study. ^1^ Ineligible due to: no longer being resident in Barwon region (n=12), <32 weeks of pregnancy (n=8), serious illness in first few days of life (n=7), major congenital disease (n=5), stillbirth (n=5), miscarriage (n=2), or cord blood stored privately (n=2). ^2^ Loss to follow-up defined as missing two consecutive reviews. *8-OHdG 8-hydroxy-2-deoxyguanosine; 8-OHGua 8-hydroxyguanosine; n sample; N total population.*

**Box S1.** Details on the experimental methods and materials for the urinary OS measurements by liquid chromatography-mass spectrometry (LC-MS/MS).

1. **Experimental methods and materials**
   1. ***Materials***

Organic solvents were all Optima-LCMS grade (ThermoFischer Scientific; Malaga, WA, Australia). LC-MS grade water was generated from a Milli-Q® IQ 7000 water purification system (Merck Millipore; Burlington Massachusetts, USA). Analytical reference standards, 8-hydroxyguanosine (8-OHGua) and 8-hydroxy-2’-deoxyguanosine (8-OHdG) and stable isotope labelled internal standards (ISTD) 8-hydroxyguanosine-^13^C^15^N_2_ (8-OHGua-^13^C^15^N_2_)(Cayman Chemical; Ann Arbour, MI, USA) and 8-hydroxy-2’-deoxyguanosine-^13^C^15^N_2_ (8-OHdG-^13^C^15^N_2_) (Toronto Research Chemicals; Toronto, ON, Canada) were used.

- 1. ***Preparation of standards and samples***

Calibration standards were prepared from a working stock solution at 50 ng/mL in methanol to generate concentrations at 0.4, 0.9, 1.5, 3, 7.5, 15, 30 and 50 ng/mL. Quality control samples were prepared at concentrations of 4, 8, 16 and 40 ng/mL. Stable isotope labelled internal standards were combined to prepare a working solution at a concentrations 8-OHGua-^13^C^15^N_2_ (20 ng/mL) and 8-OHdG-^13^C^15^N_2_ (50 ng/mL).

Urine samples were thawed at 4 °C before aliquoting 40 µL to a 96-well plate. 40 µL of ISTD and 500 µL water was added to each sample well and mixed prior to micro-solid phase extraction (SPE) treatment. The Strata-X micro-SPE well plate was conditioned with 200 μL methanol, followed by 200 μL water, each eluted at 100 rcf for 2 min at 10 °C. Samples were transferred to conditioned Strata-X polymeric micro-SPE plates (Phenomenex; Lane Cove West, NSW, Australia) and centrifuged at 100 rcf for 2 mins at 10 °C. 30 µL methanol was added to the SPE well plate to elute the analytes of interest with centrifugation at 150 rcf for 5 mins at 10 °C. 30 µL water is added to the eluted sample, followed by heat-sealing of well-plate and mixing at 1200 rpm at 10 °C for 10 min. The sample well-plate is then subjected to LC-MS/MS analysis immediately.

- 1. ***Liquid chromatography-mass spectrometry (LC-MS/MS)***

Chromatographic separation was performed using an ExionLC^TM^ system (SCIEX; Framingham, MA, USA), comprised of a binary ultra-high pressure gradient pump with degasser, a thermostated autosampler and column oven. Reversed-phase separation was performed using a Kinetex C_8_ 2.6 μm, 2.1 x 150 mm column (Phenomenex, NSW, Australia) at 40°C. Mobile phase A was water containing 0.1 % formic acid and mobile phase B was methanol containing 0.1 % formic acid. The flow rate was 0.4 mL/min with gradient elution starting at 5% B, increasing to 95% B at 18 min, holding at 95% B until 20 min, then returning to 5% B for a 2 min re-equilibration for a total cycle time of 22 min. The injection volume was 5 μL. The weak needle wash was isopropanol/water (10/90, v/v) and the strong needle wash was isopropanol.

Mass spectrometry detection with electrospray ionisation was performed using a QTRAP 6500+ system (SCIEX; Framingham, MA, USA), operated in low mass mode with polarity switching. The following mass spectrometer settings were used; capillary voltage 5200 V at positive ion mode, temperature 520°C; curtain gas 30 psi; ion source gas 1, 85 psi; ion source gas 2, 50 psi. Nitrogen was used as the curtain gas, ion source gas and collision gas. Time-scheduled multiple reaction monitoring (MRM) was used for data acquisition, details of which are provided in the Supplementary Table S1. Data were acquired using Analyst®1.7.1 and analysed using SCIEX OS Analytics 1.7.0 software (SCIEX, MA, USA).

**Table S1.** The LC-MS/MS parameters used in the experimental methods.

| **Analyte** | **Precursor (m/z)** | **Product**  **(m/z)** | **RT (min)** | **Polarity** | **DP** | **EP** | **CE** | **CXP** | **ISTD** |
| --- | --- | --- | --- | --- | --- | --- | --- | --- | --- |
| 8-OHGua | 300 | 168 | 1.60 | +ve | 40 | 10 | 21 | 20 | 8-OHGua-^13^C^15^N_3_ |
| 8-OHdG | 284 | 168 | 2.09 | +ve | 40 | 10 | 17 | 8 | 8-OHdG-^13^C^15^N_3_ |
| 8-OHGua-^13^C^15^N_3_ | 303 | 171 | 1.60 | +ve | 40 | 10 | 21 | 20 | - |
| 8-OHdG-^13^C^15^N_3_ | 287 | 171 | 2.09 | +ve | 40 | 10 | 19 | 8 | - |

*8-OHdG 8-hydroxy-2-deoxyguanosine; 8-OHdG-^13^C^15^N_2_ 8-hydroxy-2’-deoxyguanosine-^13^C^15^N_2_; 8-OHGua 8-hydroxyguanosine; 8-OHGua-^13^C^15^N_2_ 8-hydroxyguanosine-^13^C^15^N_2_; CE collision energy; CXP collision cell exit potential; DP declustering potential; EP entrance potential; ISTD internal standard; LC-MS/MS liquid chromatography-mass spectrometry; m/z mass-to-charge ratio; RT retention time.*

**Table S2.** Inter-batch precision in the measurement of urinary OS biomarkers.

|  | **Coefficient of variation (%)** | |
| --- | --- | --- |
| **QC concentration (ng/mL)** | **8-OHGua** | **8-OHdG** |
| 4 | 7.59 | 7.02 |
| 8 | 8.18 | 9.36 |
| 16 | 6.03 | 6.47 |
| 40 | 8.31 | 7.15 |

*QC quality control; 8-OHdG 8-hydroxy-2-deoxyguanosine; 8-OHGua 8-hydroxyguanosine; OS oxidative stress.*

**Table S3.** Extended sample characteristics**.**

|  |  | **2 years (n=675)** | |  | **4 years (n=791)** | |
| --- | --- | --- | --- | --- | --- | --- |
| **Factors** | | N | n (%) or  mean [SD] |  | N | n (%) or  mean [SD] |
| **Demographic** | |  |  |  |  |  |
| Paternal age (<25 years) | | 642 | 18 (2.8) |  | 749 | 25 (3.3) |
| Paternal education (lower)^a^ | | 658 | 392 (59.6) |  | 773 | 473 (61.2) |
| Maternal COB (not Australia) | | 675 | 607 (89.9) |  | 791 | 707 (89.4) |
| Maternal ancestry (Caucasian) | | 673 | 620 (92.1) |  | 789 | 725 (91.9) |
| Paternal ancestry (Caucasian) | | 672 | 606 (90.2) |  | 788 | 707 (89.7) |
|  |  |  |  |  |  |  |
| **Household composition** | |  |  |  |  |  |
| Birth spacing, weeks ^b^ | | 388 | 4.8 [4.3] |  | 447 | 4.9 [4.3] |
| Number of older siblings ^c^ | | 673 | 83 (12.3) |  | 789 | 98 (12.4) |
|  |  |  |  |  |  |  |
| **Prenatal** | |  |  |  |  |  |
| Pre-pregnancy BMI, kg/m^2^ | | 610 | 25.3 [5.5] |  | 696 | 25.3 [5.4] |
| Folate levels in RBC, nmol/L | | 461 | 0.0 [1.0] |  | 547 | 0.0 [1.0] |
| Folate supplement use | | 653 | 269.3 [91.9] |  | 761 | 271.7 [92.6] |
| Omega-3 supplement use, g/day | | 647 | 0.4 [0.5] |  | 753 | 0.4 [0.5] |
| Vitamin D supplement use | | 660 | 605 (91.7) |  | 767 | 701 (91.4) |
| Folic acid supplement use | | 660 | 650 (98.5) |  | 767 | 753 (98.2) |
| Iron supplement use | | 660 | 594 (90.0) |  | 767 | 694 (90.5) |
| Hayfever | | 659 | 428 (64.9) |  | 774 | 515 (66.5) |
| Asthma | | 665 | 335 (50.4) |  | 781 | 389 (49.8) |
| Antibiotic use | | 675 | 111 (16.4) |  | 791 | 135 (17.1) |
| Antidepressant use | | 675 | 32 (4.7) |  | 791 | 35 (4.4) |
| SSRI / SNRI use | | 651 | 27 (4.1) |  | 761 | 32 (4.2) |
| Paracetamol use | | 673 | 527 (78.3) |  | 790 | 627 (79.4) |
| Edinburgh Depression Scale: | | 508 |  |  | 575 |  |
|  | Low risk (<10 ) |  | 441 (86.8) |  |  | 490 (85.2) |
|  | Moderate risk (10-12) |  | 46 (9.1) |  |  | 58 (10.1) |
|  | High risk (>12) |  | 21 (4.1) |  |  | 27 (4.7) |
| Alcohol consumption | | 656 | 326 (49.7) |  | 763 | 398 (52.2) |
| Marijuana use | | 671 | 10 (1.5) |  | 784 | 9 (1.1) |
| Chlorine use | | 488 | 251 (51.4) |  | 550 | 280 (50.9) |
|  |  |  |  |  |  |  |
| **Perinatal** | |  |  |  |  |  |
| Child's sex | | 675 | 356 (52.7) |  | 791 | 411 (52.0) |
| Mode of birth: | | 675 |  |  | 791 |  |
|  | Unassisted vaginal birth |  | 318 (47.1) |  |  | 379 (47.9) |
|  | Instrumental vaginal delivery |  | 141 (20.9) |  |  | 155 (19.6) |
|  | Elective caesarean section |  | 119 (17.6) |  |  | 143 (18.1) |
|  | Emergency caesarean section |  | 97 (14.4) |  |  | 114 (14.4) |
| Prematurity | | 675 | 28 (4.1) |  | 791 | 35 (4.4) |
| Fetal macrosomia | | 675 | 54 (8.0) |  |  | 68 (8.6) |
| Child vitamin D in RBC, nmol/L | | 219 | 53.4 [18.8] |  | 245 | 53.3 [18.3] |
| Maternal vitamin D in RBC, nmol/L | | 289 | 87.1 [23.6] |  | 320 | 87.0 [24.1] |
| Air pollutant: | |  |  |  |  |  |
|  | NO_2_, ppb | 675 | 5.4 [1.9] |  | 791 | 5.5 [2.1] |
|  | PM_2.5_, μg/m^3^ | 675 | 7.6 [1.0] |  | 791 | 7.7 [.0] |
|  |  |  |  |  |  |  |
| **Postnatal** | |  |  |  |  |  |
| Breastfeeding at 4 weeks | | 675 | 606 (89.8) |  | 789 | 702 (89.0) |
| Breastfeeding duration (>6 months) | | 672 | 445 (66.2) |  | 784 | 510 (65.1) |
| Maternal smoking at 6 months | | 650 | 28 (4.3) |  | 756 | 34 (4.5) |
| Paternal smoking at 6 months | | 650 | 73 (11.2) |  | 756 | 94 (12.4) |
| Child's weight at 12 months, g | | 647 | 10.1 [1.2] |  | 742 | 10.1 [1.2] |
| Child SHS exposure at 12 months | | 661 | 37 (5.6) |  | 771 | 43 (5.6) |
| Home heating: ^d^ | |  |  |  |  |  |
|  | Electric convection heater | 666 | 27 (4.1) |  | 618 | 26 (4.2) |
|  | RCA conditioning unit | 666 | 108 (16.2) |  | 618 | 96 (15.5) |
|  | Oil / kerosene / diesel heater | 666 | 10 (1.5) |  | 618 | 9 (1.5) |
|  | Central heating (ducted air) | 666 | 169 (25.4) |  | 618 | 153 (24.8) |
|  | Gas heater without a flue ^e^ | 666 | 13 (2.0) |  | 618 | 13 (2.1) |
| Floor covering: ^d^ | |  |  |  |  |  |
|  | Linoleum / vinyl | 666 | 12 (1.8) |  | 618 | 13 (2.1) |
|  | Carpet | 666 | 142 (21.3) |  | 618 | 132 (21.4) |
|  | Rug | 666 | 96 (14.4) |  | 618 | 87 (14.1) |
|  | Timber / wooden | 666 | 148 (22.2) |  | 618 | 137 (22.2) |
| Cook top: gas type ^d^ | | 666 | 277 (41.6) |  | 618 | 254 (41.1) |
| Oven type: gas type ^d^ | | 666 | 86 (12.9) |  | 618 | 75 (12.1) |

^a^ Paternal education (lower indicate no university degree). ^b^ Birth spacing with the oldest sibling. ^c^ Number of older siblings aged two to six years. ^d^ Home environment factors collected at two years are stable from pregnancy exposure. ^e^ Gas heater without a flue (including portable gas heaters). *BMI body mass index; COB country of birth; NO_2_ nitric dioxide; ppb parts per billion; RBC red blood cells; RCA recycled concrete aggregate; SHS secondhand smoke; SSRI selective serotonin reuptake inhibitor; SNRI serotonin and norepinephrine reuptake inhibitors.*

**Table S4.** Associations between early life factors with (i) maternal OS biomarkers at 36 weeks of pregnancy; and (ii) child total EBP at ages 2 and 4 years.

|  |  | **Maternal OS Biomarkers** | | | | |  | **Child Total EBP** | | | | |
| --- | --- | --- | --- | --- | --- | --- | --- | --- | --- | --- | --- | --- |
|  |  | **8-OHGua, ng/mL** |  |  | **8-OHdG, ng/mL** | |  | **2 years** | |  | **4 years** | |
| **Early life factors** | | *β* (95% CI) * | *P* |  | *β* (95% CI) * | *P* |  | *β* (95% CI) **^†^** | *P* |  | *β* (95% CI) **^†^** | *P* |
| **Demographic** | |  |  |  |  |  |  |  |  |  |  |  |
| Paternal age (<25 years) | | 0.35 (-0.04, 0.74) | 0.08 |  | 0.06 (-0.21, 0.34) | 0.64 |  | **6.30 (2.58, 10.01)** | **0.001** |  | **2.43 (0.54, 4.32)** | **0.01** |
| Paternal education (lower)^a^ | | 0.05 (-0.09, 0.20) | 0.48 |  | -0.002 (-0.12, 0.11) | 0.97 |  | 0.63 (-0.74, 2.01) | 0.37 |  | **1.23 (0.60, 1.87)** | **<0.0001** |
| Maternal COB (not Australia) | | -0.03 (-0.27, 0.21) | 0.79 |  | 0.09 (-0.10, 0.27) | 0.37 |  | -2.14 (-4.38, 0.09) | 0.06 |  | -0.96 (-1.98, 0.05) | 0.06 |
| Maternal ancestry (Caucasian) | | -0.01 (-0.27, 0.25) | 0.94 |  | -0.007 (-0.18, 0.17) | 0.94 |  | **-3.50 (-5.89, -1.10)** | **0.004** |  | -0.31 (-1.46, 0.85) | 0.60 |
| Paternal ancestry (Caucasian) | | -0.16 (-0.35, 0.02) | 0.08 |  | -0.07 (-0.25, 0.11) | 0.47 |  | **-2.49 (-4.54, -0.43)** | **0.02** |  | -0.23 (-1.22, 0.76) | 0.65 |
|  | |  |  |  |  |  |  |  |  |  |  |  |
| **Household composition** | |  |  |  |  |  |  |  |  |  |  |  |
| Birth spacing, weeks ^b^ | | 0.008 (-0.01, 0.03) | 0.49 |  | 0.004 (-0.01, 0.02) | 0.63 |  | 0.04 (-0.17, 0.24) | 0.71 |  | **0.14 (0.02, 0.27)** | **0.02** |
| Number of older siblings ^c^ | | -0.07 (-0.29, 0.16) | 0.57 |  | -0.09 (-0.26, 0.08) | 0.29 |  | 0.59 (-1.51, 2.70) | 0.58 |  | **-1.30 (-2.17, -0.43)** | **0.003** |
|  | |  |  |  |  |  |  |  |  |  |  |  |
| **Prenatal** | |  |  |  |  |  |  |  |  |  |  |  |
| Pre-pregnancy BMI, kg/m^2^ | | 0.01 (-0.005, 0.03) | 0.15 |  | 0.01 (-0.002, 0.02) | 0.12 |  | 0.09 (-0.04, 0.21) | 0.17 |  | **0.07 (-0.0001, 0.13)** | **0.05** |
| Folate levels in RBC, nmol/L | | 0.09 (-0.008, 0.19) | 0.07 |  | **0.10 (0.03, 0.17)** | **0.01** |  | -0.09 (-0.92, 0.73) | 0.82 |  | -0.19 (-0.60, 0.21) | 0.35 |
| Folate supplement use | | -0.0004 (-0.001, 0.001) | 0.41 |  | -0.0003 (-0.001, 0.0004) | 0.42 |  | -0.0004 (-0.008, 0.007) | 0.92 |  | **-0.004 (-0.008, -0.0004)** | **0.03** |
| Omega-3 supplement use, g/day | | 0.13 (-0.004, 0.26) | 0.06 |  | -0.03 (-0.15, 0.09) | 0.65 |  | -0.13 (-1.51, 1.26) | 0.86 |  | 0.13 (-0.50, 0.76) | 0.69 |
| Vitamin D supplement use | | 0.03 (-0.20, 0.26) | 0.81 |  | 0.16 (-0.04, 0.35) | 0.11 |  | -0.73 (-3.02, 1.56) | 0.53 |  | -0.41 (-1.56, 0.74) | 0.48 |
| Folic acid supplement use | | -0.30 (-0.61, 0.007) | 0.06 |  | -0.02 (-0.40, 0.36) | 0.93 |  | 2.32 (-2.91, 7.54) | 0.38 |  | -0.74 (-3.54, 2.05) | 0.60 |
| Iron supplement use | | -0.15 (-0.35, 0.04) | 0.13 |  | -0.02 (-0.19, 0.16) | 0.87 |  | -0.22 (-2.37, 1.93) | 0.84 |  | -0.47 (-1.59, 0.65) | 0.41 |
| Hayfever | | -0.07 (-0.21, 0.08) | 0.36 |  | -0.10 (-0.21, 0.02) | 0.10 |  | 0.78 (-0.62, 2.18) | 0.28 |  | 0.38 (-0.30, 1.05) | 0.28 |
| Asthma | | -0.07 (-0.22, 0.08) | 0.35 |  | -0.01 (-0.12, 0.09) | 0.80 |  | 0.60 (-0.75, 1.94) | 0.38 |  | **0.73 (0.09, 1.36)** | **0.02** |
| Antibiotic use | | -0.005 (-0.21, 0.19) | 0.96 |  | -0.03 (-0.18, 0.12) | 0.71 |  | 0.94 (-0.85, 2.73) | 0.30 |  | 0.14 (-0.70, 0.98) | 0.74 |
| Antidepressant use | | 0.06 (-0.29, 0.42) | 0.72 |  | 0.05 (-0.15, 0.26) | 0.62 |  | 0.85 (-2.45, 4.15) | 0.61 |  | **4.12 (2.19, 6.06)** | **<0.0001** |
| SSRI / SNRI use | | 0.06 (-0.34, 0.46) | 0.76 |  | 0.02 (-0.21, 0.24) | 0.89 |  | 1.67 (-2.05, 5.38) | 0.38 |  | **3.77 (1.76, 5.78)** | **<0.0001** |
| Paracetamol use | | 0.05 (-0.13, 0.22) | 0.59 |  | -0.03 (-0.16, 0.11) | 0.71 |  | -0.23 (-1.83, 1.38) | 0.78 |  | 0.66 (-0.11, 1.43) | 0.09 |
| Edinburgh Depression Scale, score: | |  |  |  |  |  |  |  |  |  |  |  |
|  | Low risk (<10) | Reference |  |  | Reference |  |  | Reference |  |  | Reference |  |
|  | Moderate risk (10-12) | -0.03 (-0.21, 0.14) | 0.71 |  | -0.02 (-0.14, 0.11) | 0.80 |  | 1.29 (-0.24, 2.82) | 0.10 |  | **1.24 (0.52, 1.97)** | **0.001** |
|  | High risk (>12) | -0.006 (-0.03, 0.02) | 0.64 |  | -0.002 (-0.02, 0.01) | 0.74 |  | -0.19 (-0.61, 0.23) | 0.37 |  | **1.42 (0.06, 2.77)** | **0.04** |
|  | *P trend* |  |  |  |  |  |  |  |  |  |  |  |
| Alcohol consumption | | -0.11 (-0.25, 0.04) | 0.15 |  | **-0.14 (-0.25, -0.03)** | **0.01** |  | -0.48 (-1.82, 0.87) | 0.49 |  | -0.42 (-1.07, 0.23) | 0.21 |
| Marijuana use | | -0.49 (-0.99, 0.02) | 0.06 |  | -0.21 (-0.56, 0.13) | 0.22 |  | 4.92 (-0.82, 10.66) | 0.09 |  | 1.71 (-1.37, 4.79) | 0.28 |
| Air freshener use | | 0.13 (-0.02, 0.29) | 0.10 |  | 0.06 (-0.06, 0.18) | 0.33 |  | **1.94 (0.32, 3.55)** | **0.02** |  | **1.17 (0.41, 1.94)** | **0.003** |
| Chlorine use | | 0.08 (-0.08, 0.23) | 0.32 |  | 0.01 (-0.11, 0.13) | 0.85 |  | 1.51 (-0.07, 3.08) | 0.06 |  | 0.44 (-0.32, 1.20) | 0.26 |
|  | |  |  |  |  |  |  |  |  |  |  |  |
| **Perinatal** | |  |  |  |  |  |  |  |  |  |  |  |
| Child's sex | | -0.06 (-0.21, 0.08) | 0.40 |  | -0.05 (-0.16, 0.05) | 0.33 |  | 0.90 (-0.44, 2.23) | 0.19 |  | **0.92 (0.28, 1.55)** | **0.005** |
| Mode of birth: | |  |  |  |  |  |  |  |  |  |  |  |
|  | Unassisted vaginal birth | Reference |  |  | Reference |  |  | Reference |  |  | Reference |  |
|  | Instrumental vaginal delivery | -0.04 (-0.23, 0.15) | 0.69 |  | -0.08 (-0.22, 0.06) | 0.28 |  | 0.90 (-0.85, 2.65) | 0.31 |  | 0.47 (-0.27, 1.42) | 0.33 |
|  | Elective caesarean section | -0.05 (-0.26, 0.15) | 0.61 |  | -0.11 (-0.26, 0.05) | 0.17 |  | -1.08 (-2.89, 0.73) | 0.24 |  | -0.55 (-1.41, 0.31) | 0.21 |
|  | Emergency caesarean section | -0.01 00(-0.24, 0.21) | 0.92 |  | -0.07 (-0.23, 0.09) | 0.38 |  | 0.85 (-1.15, 2.84) | 0.41 |  | 0.36 (-0.54, 1.26) | 0.43 |
|  | *P trend* |  | *0.74* |  |  | *0.18* |  |  | *0.89* |  |  | *0.98* |
| Prematurity | | -0.35 (-1.03, 0.34) | 0.32 |  | **-0.5 (-0.88, -0.13)** | **0.008** |  | 1.15 (-2.54, 4.83) | 0.54 |  | 1.24 (-0.65, 3.14) | 0.20 |
| Apgar score at 5 minutes | | **-0.09 (-0.16, -0.03)** | **0.002** |  | **-0.06 (-0.11, -0.003)** | **0.04** |  | **-0.95 (-1.69, -0.22)** | **0.01** |  | -0.3 (-0.69, 0.08) | 0.12 |
| Fetal macrosomia | | **0.27 (0.02, 0.51)** | **0.03** |  | 0.08 (-0.09, 0.25) | 0.37 |  | 1.96 (-0.50, 4.42) | 0.12 |  | 0.89 (-0.34, 2.12) | 0.16 |
| Child vitamin D in RBC, nmol/L | | 0.005 (-0.002, 0.01) | 0.16 |  | 0.004 (-0.001, 0.01) | 0.11 |  | 0.02 (-0.04, 0.09) | 0.51 |  | -0.01 (-0.04, 0.02) | 0.38 |
| Maternal vitamin D in RBC, nmol/L | | -0.001 (-0.006, 0.004) | 0.73 |  | 0.001 (-0.003, 0.005) | 0.51 |  | -0.008 (-0.05, 0.03) | 0.71 |  | **-0.02 (-0.04, -0.005)** | **0.01** |
| Air pollutant: | |  |  |  |  |  |  |  |  |  |  |  |
|  | PM_2.5_, μg/m^3^ | **0.10 (0.03, 0.16)** | **0.003** |  | 0.04 (-0.02, 0.09) | 0.17 |  | 0.43 (-0.28, 1.14) | 0.23 |  | -0.03 (-0.35, 0.29) | 0.85 |
|  | NO_2_, ppb | 0.02 (-0.02, 0.06) | 0.26 |  | 0.001 (-0.02, 0.03) | 0.91 |  | 0.09 (-0.24, 0.42) | 0.60 |  | -0.02 (-0.18, 0.14) | 0.84 |
|  |  |  |  |  |  |  |  |  |  |  |  |  |
| **Postnatal** | |  |  |  |  |  |  |  |  |  |  |  |
| Breastfeeding at 4 weeks | | **-0.2 (-0.41, 0.004)** | **0.05** |  | 0.02 (-0.16, 0.19) | 0.85 |  | -0.46 (-2.59, 1.66) | 0.67 |  | **-1.50 (-2.61, -0.38)** | **0.009** |
| Breastfeeding duration (>6 months) | | -0.05 (-0.20, 0.11) | 0.54 |  | -0.01 (-0.13, 0.11) | 0.87 |  | -0.05 (-1.46, 1.36) | 0.94 |  | **-0.82 (-1.49, -0.14)** | **0.02** |
| Maternal smoking at 6 months | | 0.29 (-0.09, 0.68) | 0.14 |  | **0.32 (0.05, 0.59)** | **0.02** |  | 1.07 (-2.22, 4.36) | 0.52 |  | 1.12 (-0.36, 2.59) | 0.14 |
| Paternal smoking at 6 months | | **0.32 (0.08, 0.55)** | **0.008** |  | 0.14 (-0.03, 0.31) | 0.11 |  | **2.61 (0.53, 4.70)** | **0.01** |  | **1.52 (0.58, 2.45)** | **0.001** |
| Child's weight at 12 months | | 0.02 (-0.04, 0.08) | 0.46 |  | -0.01 (-0.05, 0.03) | 0.66 |  | 0.34 (-0.28, 0.95) | 0.29 |  | 0.20 (-0.09, 0.49) | 0.17 |
| Child SHS exposure at 12 months | | **0.33 (0.02, 0.65)** | **0.04** |  | 0.18 (-0.09, 0.44) | 0.19 |  | **5.29 (2.27, 8.3)** | **0.001** |  | **2.62 (1.26, 3.98)** | **<0.0001** |
| Home heating at 2 years: ^d^ | |  |  |  |  |  |  |  |  |  |  |  |
|  | Electric convection heater | **0.3 (0.01, 0.6)** | **0.05** |  | 0.14 (-0.14, 0.41) | 0.33 |  | 0.25 (-3.10, 3.60) | 0.88 |  | -0.59 (-2.27, 1.1) | 0.49 |
|  | RCA conditioning unit | 0.17 (-0.03, 0.37) | 0.09 |  | 0.13 (-0.03, 0.29) | 0.11 |  | 0.08 (-1.75, 1.91) | 0.93 |  | 0.86 (-0.16, 1.88) | 0.10 |
|  | Oil / kerosene / diesel heater | -0.21 (-0.93, 0.51) | 0.57 |  | -0.2 (-0.72, 0.32) | 0.45 |  | **4.90 (0.54, 9.26)** | **0.03** |  | **4.30 (0.09, 8.52)** | **0.05** |
|  | Central heating (ducted air) | **0.19 (0.00, 0.38)** | **0.05** |  | 0.01 (-0.14, 0.16) | 0.89 |  | **-2.73 (-4.28, -1.18)** | **0.001** |  | -0.55 (-1.35, 0.25) | 0.18 |
|  | Gas heater without a flue ^e^ | -0.24 (-0.93, 0.45) | 0.49 |  | 0.09 (-0.34, 0.51) | 0.69 |  | 2.45 (-3.55, 8.46) | 0.42 |  | **2.94 (0.27, 5.61)** | **0.03** |
| Living room flooring at 2 years: ^d^ | |  |  |  |  |  |  |  |  |  |  |  |
|  | Linoleum / vinyl | -0.24 (-0.71, 0.23) | 0.32 |  | 0.03 (-0.35, 0.42) | 0.86 |  | -3.14 (-9.61, 3.34) | 0.34 |  | 3.43 (-0.51, 7.37) | 0.09 |
|  | Carpet | 0.08 (-0.11, 0.28) | 0.40 |  | -0.08 (-0.23, 0.08) | 0.33 |  | 0.70 (-1.02, 2.42) | 0.42 |  | **1.07 (0.23, 1.90)** | **0.01** |
|  | Rug | 0.10 (-0.13, 0.32) | 0.40 |  | 0.13 (-0.05, 0.31) | 0.15 |  | **-2.8 (-4.59, -1.00)** | **0.002** |  | **-1.01 (-2.02, 0.00)** | **0.05** |
|  | Timber / wooden | 0.14 (-0.06, 0.34) | 0.16 |  | 0.09 (-0.06, 0.25) | 0.24 |  | **-2.03 (-3.66, -0.40)** | **0.01** |  | **-1.13 (-1.95, -0.31)** | **0.01** |
| Cook top at 2 years: gas ^d^ | | **0.17 (0.00, 0.33)** | **0.05** |  | 0.09 (-0.04, 0.22) | 0.16 |  | -1.20 (-2.58, 0.18) | 0.09 |  | -0.42 (-1.14, 0.31) | 0.26 |
| Oven type at 2 years: gas ^d^ | | **0.23 (0.02, 0.44)** | **0.04** |  | 0.07 (-0.13, 0.26) | 0.49 |  | 0.44 (-1.51, 2.39) | 0.66 |  | -0.63 (-1.71, 0.45) | 0.25 |

^*^ Minimally adjusted for child’s sex and gestational age at the time of urine collection. ^†^ Minimally adjusted for child’s sex and child’s age at the time of behavioural assessment. ^a^ Paternal education (lower indicate no university degree). ^b^ Birth spacing with the oldest sibling. ^c^ Number of older siblings aged two to six years. ^d^ Home environment factors collected at two years are stable from pregnancy exposure. ^e^ Gas heater without a flue (including portable gas heaters). * Factors indicate there was consistently associated with both OS biomarkers. *8-OHdG 8-hydroxy-2-deoxyguanosine; 8-OHGua 8-hydroxyguanosine; AUD Australian dollars; BMI body mass index; COB country of birth; EBP emotional and behavioural problems; ng/mL per 1 nanograms per millilitre; NO_2_ nitric dioxide; ppb parts per billion; PM_2.5_ particulate matter 2.5; RBC red blood cells; RCA recycled concrete aggregate; SHS secondhand smoke; SSRI selective serotonin reuptake inhibitor; SNRI serotonin and norepinephrine reuptake inhibitors; μg/m^3^ one millionth of a gram of a contaminant per cubic meter of ambient air.*

**Table S5.** Associations between key early life factors and EBP at ages 2 years and 4 years, mediated by maternal 8-OHGua biomarker levels at 36 weeks during pregnancy, adjusted for relevant covariates.

|  |  |  |  | **Child EBP Outcomes** | | | | | | | | |
| --- | --- | --- | --- | --- | --- | --- | --- | --- | --- | --- | --- | --- |
|  |  |  |  | **2 years** | | | |  | **4 years** | | | |
|  | **Maternal 8-OHGua** | |  | **Total EBP** | | **DSM-5 DP** | |  | **Total EBP** | | **ES** | |
| **Factors** | *β* (95% CI) | *P* | **Effects** | *β* (95% CI) | *P* | *β* (95% CI) | *P* |  | *β* (95% CI) | *P* | *β* (95% CI) | *P* |
| Maternal education (lower) | **0.17 (0.02, 0.32)** | **0.02** | Total Effect | 0.82 (-0.75, 2.29) | 0.26 | 0.00 (-0.69, 0.66) | 1.00 |  | **1.19 (0.46, 1.87)** | **<0.0001** | **0.40 (0.14, 0.64)** | **0.002** |
|  |  |  | Direct Effect | 0.72 (-0.84, 2.19) | 0.33 | -0.06 (-0.74, 0.58) | 0.84 |  | **1.11 (0.40, 1.80)** | **<0.0001** | **0.37 (0.12, 0.62)** | **0.004** |
|  |  |  | Indirect Effect | 0.11 (-0.02, 0.31) | 0.12 | ***0.07 (0.00, 0.17)*** | ***0.04*** |  | ***0.07 (0.003, 0.18)*** | ***0.04*** | **0.02 (0.00, 0.06)** | **0.04** |
|  |  |  | % Mediated | 0.09 (-0.76, 1.43) | 0.34 | 0.01 (-3.24, 3.87) | 0.98 |  | **0.06 (0.002, 0.20)** | **0.04** | **0.06 (0.00, 0.21)** | **0.05** |
|  |  |  |  |  |  |  |  |  |  |  |  |  |
| Residential SED (lowest tertile) | **0.22 (0.06, 0.38)** | **0.006** | Total Effect | 0.59 (-1.06, 2.16) | 0.46 | **0.74 (0.03, 1.42)** | **0.04** |  | 0.69 (-0.08, 1.44) | 0.07 | 0.16 (-0.11, 0.42) | 0.21 |
|  |  |  | Direct Effect | 0.45 (-1.19, 2.05) | 0.58 | 0.66 (-0.05, 1.36) | 0.06 |  | 0.6 (-0.17, 1.35) | 0.11 | 0.13 (-0.14, 0.40) | 0.31 |
|  |  |  | Indirect Effect | 0.14 (-0.04, 0.39) | 0.13 | ***0.08 (0.00, 0.19)*** | ***0.04*** |  | ***0.09 (0.009, 0.21)*** | ***0.02*** | **0.03 (0.00, 0.07)** | **0.03** |
|  |  |  | % Mediated | 0.1 (-1.88, 2.01) | 0.53 | 0.09 (-0.04, 0.51) | 0.09 |  | 0.12 (-0.29, 0.76) | 0.09 | 0.15 (-1.30, 1.57) | 0.23 |
|  |  |  |  |  |  |  |  |  |  |  |  |  |
| Prescription medication use * | **0.16 (0.01, 0.30)** | **0.04** | Total Effect | 0.13 (-1.37, 1.55) | 0.89 | 0.07 (-0.58, 0.7) | 0.84 |  | **1.06 (0.34, 1.75)** | **<0.0001** | **0.35 (0.10, 0.59)** | **0.006** |
|  |  |  | Direct Effect | 0.08 (-1.4, 1.49) | 0.94 | 0.04 (-0.61, 0.65) | 0.92 |  | **0.99 (0.28, 1.66)** | **0.006** | **0.33 (0.08, 0.56)** | **0.008** |
|  |  |  | Indirect Effect | 0.05 (-0.04, 0.21) | 0.36 | 0.03 (-0.02, 0.12) | 0.30 |  | ***0.07 (0.002, 0.17)*** | ***0.04*** | **0.02 (0.00, 0.06)** | **0.05** |
|  |  |  | % Mediated | 0.007 (-1.51, 1.1) | 0.93 | 0.02 (-2.27, 2.35) | 0.87 |  | **0.06 (0.001, 0.23)** | **0.04** | 0.06 (0.00, 0.25) | 0.06 |
|  |  |  |  |  |  |  |  |  |  |  |  |  |
| Tobacco  smoking * **^†^** | **0.31 (0.09, 0.53)** | **0.006** | Total Effect | **3.03 (0.57, 5.34)** | **0.02** | 1.01 (-0.08, 2.03) | 0.06 |  | 1.10 (-0.05, 2.20) | 0.06 | 0.05 (-0.36, 0.43) | 0.81 |
|  |  |  | Direct Effect | **2.76 (0.26, 5.18)** | **0.04** | 0.83 (-0.27, 1.89) | 0.11 |  | 0.96 (-0.19, 2.06) | 0.10 | 0.00 (-0.41, 0.38) | 0.96 |
|  |  |  | Indirect Effect | 0.27 (-0.09, 0.71) | 0.16 | ***0.18 (0.02, 0.40)*** | ***0.02*** |  | ***0.15 (0.02, 0.34)*** | ***0.01*** | **0.05 (0.01, 0.12)** | **0.01** |
|  |  |  | % Mediated | 0.08 (-0.05, 0.41) | 0.17 | 0.17 (-0.33, 1.12) | 0.08 |  | 0.12 (-0.24, 0.69) | 0.07 | 0.12 (-3.50, 4.88) | 0.81 |
|  |  |  |  |  |  |  |  |  |  |  |  |  |
| SHS  exposure * **^†^** | **0.19 (0.003, 0.38)** | **0.05** | Total Effect | 0.75 (-1.63, 3.03) | 0.51 | 0.44 (-0.61, 1.46) | 0.38 |  | **1.24 (0.23, 2.22)** | **0.01** | 0.15 (-0.21, 0.49) | 0.39 |
|  |  |  | Direct Effect | 0.58 (-1.81, 2.9) | 0.61 | 0.34 (-0.72, 1.37) | 0.51 |  | **1.14 (0.11, 2.13)** | **0.04** | 0.11 (-0.25, 0.46) | 0.52 |
|  |  |  | Indirect Effect | 0.17 (-0.02, 0.5) | 0.09 | ***0.10 (0.00, 0.26)*** | ***0.05*** |  | ***0.09 (0.001, 0.24)*** | ***0.05*** | **0.03 (0.00, 0.09)** | **0.05** |
|  |  |  | % Mediated | 0.08 (-2.35, 2.53) | 0.55 | 0.12 (-1.68, 2.16) | 0.41 |  | 0.07 (0.00, 0.36) | 0.06 | 0.12 (-1.84, 2.76) | 0.42 |

^(*)^ Prenatal factors, if any. **^†^** Factors that were consistently associated with both OS biomarkers. Adjusted for child’s sex, gestational age at the time of urine collection and child’s age at the time of behavioural assessment. *Note.* The non-reference category for each early life variable is displayed in parentheses in the first column. Maternal education (lower indicate no university degree). *8-OHGua 8-hydroxyguanosine; DSM-5 Diagnostic and Statistical Manual of Mental Disorders, Version 5; DP Depression Problems; EBP emotional and behavioural problems; ES Emotional Symptoms; SED socioeconomic disadvantage; SHS secondhand smoke.*

**Table S6.** Associations between key early life factors and EBP at ages 2 years and 4 years, mediated by maternal 8-OHGua biomarker levels at 36 weeks during pregnancy additional adjusted for lower maternal education.

|  |  |  |  | **Child EBP outcomes** | | | | | | | | |
| --- | --- | --- | --- | --- | --- | --- | --- | --- | --- | --- | --- | --- |
|  |  |  |  | **2 years** | | | |  | **4 years** | | | |
|  | **Maternal 8-OHG** | |  | **Total EBP** | | **DSM-5 DP** | |  | **Total EBP** | | **ES at 4 years** | |
| **Factors** | *β* (95% CI) | *P* | **Effects** | *β* (95% CI) | *P* | *β* (95% CI) | *P* |  | *β* (95% CI) | *P* | *β* (95% CI) | *P* |
| Residential SED (lowest tertile) | **0.22 (0.06, 0.38)** | **0.006** | Total Effect | 0.60 (-0.96, 2.23) | 0.47 | **0.77 (0.09, 1.47)** | **0.03** |  | 0.63 (-0.11, 1.39) | 0.08 | 0.13 (-0.13, 0.4) | 0.30 |
|  |  |  | Direct Effect | 0.48 (-1.07, 2.13) | 0.55 | **0.69 (0.02, 1.41)** | **0.05** |  | 0.56 (-0.17, 1.33) | 0.12 | 0.11 (-0.15, 0.38) | 0.40 |
|  |  |  | Indirect Effect | 0.12 (-0.06, 0.36) | 0.18 | 0.07 (0.001, 0.19) | 0.06 |  | 0.07 (-0.01, 0.19) | 0.08 | 0.02 (0.001, 0.07) | 0.08 |
|  |  |  | % Mediated | 0.08 (-2.33, 1.47) | 0.57 | 0.09 (-0.03, 0.47) | 0.10 |  | 0.10 (-0.24, 0.88) | 0.15 | 0.12 (-2.17, 1.54) | 0.36 |
| Prescription medication use * | **0.16 (0.01, 0.30)** | **0.04** | Total Effect | 0.19 (-1.22, 1.65) | 0.79 | 0.06 (-0.55, 0.69) | 0.84 |  | **1.03 (0.36, 1.72)** | **0.002** | **0.34 (0.10, 0.58)** | **0.002** |
|  |  |  | Direct Effect | 0.14 (-1.29, 1.62) | 0.84 | 0.03 (-0.59, 0.67) | 0.93 |  | **0.98 (0.31, 1.68)** | **0.002** | **0.32 (0.09, 0.56)** | **0.008** |
|  |  |  | Indirect Effect | 0.05 (-0.05, 0.21) | 0.45 | 0.03 (-0.03, 0.12) | 0.38 |  | 0.06 (-0.01, 0.16) | 0.11 | 0.02 (0.001, 0.05) | 0.10 |
|  |  |  | % Mediated | 0.01 (-1.13, 1.09) | 0.90 | 0.02 (-2.12, 1.4) | 0.88 |  | 0.05 (-0.01, 0.22) | 0.11 | 0.05 (-0.01, 0.26) | 0.11 |
| Tobacco smoking * | **0.31 (0.09, 0.53)** | **0.006** | Total Effect | **2.87 (0.58, 5.32)** | **0.02** | **1.06 (0.06, 2.15)** | **0.04** |  | 0.83 (-0.26, 1.99) | 0.14 | -0.03 (-0.42, 0.37) | 0.86 |
|  |  |  | Direct Effect | **2.63 (0.29, 5.11)** | **0.04** | 0.90 (-0.13, 1.98) | 0.08 |  | 0.72 (-0.38, 1.86) | 0.21 | -0.08 (-0.46, 0.32) | 0.69 |
|  |  |  | ***Indirect Effect*** | 0.24 (-0.13, 0.69) | 0.19 | ***0.16 (0.001, 0.38)*** | ***0.05*** |  | 0.11 (0.001, 0.29) | 0.06 | 0.04 (0.001, 0.11) | 0.06 |
|  |  |  | % Mediated | 0.08 (-0.05, 0.46) | 0.20 | 0.14 (-0.07, 0.85) | 0.09 |  | 0.11 (-0.91, 1.32) | 0.18 | -0.07 (-2.79, 3.51) | 0.86 |
| SHS exposure * | **0.19 (0.003, 0.38)** | **0.05** | Total Effect | 0.72 (-1.46, 3.09) | 0.53 | 0.52 (-0.43, 1.56) | 0.30 |  | **1.02 (0.08, 2.02)** | **0.05** | 0.05 (-0.27, 0.40) | 0.77 |
|  |  |  | Direct Effect | 0.58 (-1.66, 2.93) | 0.61 | 0.44 (-0.55, 1.47) | 0.37 |  | **0.95 (-0.01, 1.97)** | **0.05** | 0.03 (-0.31, 0.38) | 0.89 |
|  |  |  | Indirect Effect | 0.14 (-0.04, 0.47) | 0.17 | 0.08 (-0.01, 0.25) | 0.10 |  | 0.07 (-0.01, 0.21) | 0.14 | 0.03 (0.001, 0.08) | 0.11 |
|  |  |  | % Mediated | 0.07 (-1.37, 1.35) | 0.60 | 0.10 (-1.36, 1.4) | 0.36 |  | 0.06 (-0.06, 0.41) | 0.18 | 0.05 (-2.12, 2.06) | 0.78 |

^(*)^ Prenatal factors, if any. *Note.* The non-reference category for each early life variable is displayed in parentheses in the first column. Adjusted for child’s sex, gestational age at the time of urine collection, child’s age at the time of behavioural assessment and maternal education. *8-OHGua 8-hydroxyguanosine; DSM-5 Diagnostic and Statistical Manual of Mental Disorders, Version 5; DP Depression Problems; EBP emotional and behavioural problems; ES Emotional Symptoms; SED socioeconomic disadvantage; SHS secondhand smoke.*

**Table S7.** Linear regression estimates for the associations between maternal OS biomarkers at 36 weeks of pregnancy and EBP at ages 2 and 4 years in models: (A) and (B) with an interaction term for OS biomarker and child’s sex; (C) with additional adjustment for maternal weight gain during pregnancy; and (D) where OS biomarkers were corrected for urine osmolality instead of specific gravity.

| **Maternal OS Biomarkers (ng/mL)** | **Child Total EBP** | | | | | | | | |
| --- | --- | --- | --- | --- | --- | --- | --- | --- | --- |
|  | **2 years** | | | |  | **4 years** | | | |
|  | *β* (95% CI) ^*^ | *P* | *β* (95% CI) **^†^** | *P* |  | *β* (95% CI) ^*^ | *P* | *β* (95% CI) | *P* |
| **A. Model with interaction term for 8-OHGua and child’s sex** | | | | | | | | |  |
| 8-OHGua (for females) | 0.38 (-0.69, 1.45) | 0.48 | 0.35 (-0.71, 1.40) | 0.52 |  | 0.43 (-0.01, 0.87) | 0.06 | 0.38 (-0.06, 0.82) | 0.09 |
| 8-OHGua (for males) | 0.82 (-0.04, 1.67) | 0.06 | 0.83 (-0.05, 1.71) | 0.06 |  | 0.41 (-0.04, 0.85) | 0.07 | 0.38 (-0.05, 0.82) | 0.08 |
| 8-OHGua $\times$ Sex(male) | 0.43 (-0.94, 1.80) | 0.53 | 0.49 (-0.88, 1.86) | 0.49 |  | -0.02 (-0.64, 0.61) | 0.95 | 0.00 (-0.62, 0.62) | 0.99 |
|  |  |  |  |  |  |  |  |  |  |
| **B. Model with interaction term for 8-OHdG and child’s sex** | | | | | | | | | |
| 8-OHdG (for females) | -0.34 (-1.61, 0.94) | 0.61 | -0.35 (-1.62, 0.93) | 0.59 |  | 0.17 (-0.43, 0.77) | 0.58 | 0.16 (-0.43, 0.75) | 0.59 |
| 8-OHdG (for males) | 0.40 (-0.83, 1.63) | 0.52 | 0.62 (-0.59, 1.83) | 0.31 |  | 0.00 (-0.61, 0.62) | 0.99 | 0.08 (-0.52, 0.67) | 0.80 |
| 8-OHdG $\times$ Sex(male) | 0.74 (-1.05, 2.52) | 0.42 | 0.97 (-0.80, 2.74) | 0.28 |  | -0.17 (-1.03, 0.69) | 0.70 | -0.08 (-0.92, 0.75) | 0.84 |
|  |  |  |  |  |  |  |  |  |  |
| **C. Models additional adjusted for maternal weight gain during pregnancy** | | | | | | | | | |
| 8-OHG | **0.87 (0.15, 1.58)** | **0.02** | **0.74 (0.03, 1.46)** | **0.04** |  | **0.49 (0.15, 0.82)** | **0.004** | **0.41 (0.06, 0.75)** | **0.02** |
| 8-OHdG | 0.70 (-0.24, 1.64) | 0.14 | **0.43 (0.10, 0.76)** | **0.01** |  | 0.27 (-0.21, 0.74) | 0.27 | 0.70 (-0.23, 1.63) | 0.14 |
|  |  |  |  |  |  |  |  |  |  |
| **D. Models where OS biomarkers were corrected for urine osmolality instead of specific gravity** | | | | | | | | |  |
| 8-OHG | 0.14 (-0.44, 0.72) | 0.63 | 0.11 (-0.47, 0.69) | 0.70 |  | **0.30 (0.04, 0.56)** | **0.03** | **0.27 (0.01, 0.53)** | **0.04** |
| 8-OHdG | -0.32 (-0.99, 0.34) | 0.34 | -0.28 (-0.93, 0.38) | 0.41 |  | 0.04 (-0.26, 0.34) | 0.80 | 0.05 (-0.24, 0.35) | 0.72 |

^*^ Minimally adjusted for child's sex, gestational age at urine collection, and child's age at the time of behavioural assessment. **^†^** Fully additionally adjusted for maternal age and household income. *8-OHdG 8-hydroxydeoxyguaosine; 8-OHGua 8-hydroxyguanosine; EBP emotional and behavioural problems; ng/mL per 1 nanograms per millilitre; OS oxidative stress.*

**Table S8.** Associations between individual child OS-related genes and maternal OS biomarkers at 36 weeks of pregnancy.

| **Child OS-related *Gene* (SNP) *** | **Minor alleles** | **Effect of SNP on gene expression** | **Maternal OS Biomarkers** | | | | | | |
| --- | --- | --- | --- | --- | --- | --- | --- | --- | --- |
|  |  |  | **8-OHGua, ng/mL** | | |  | **8-OHdG, ng/mL** | | |
|  |  |  | N | β (95% CI) ^†^ | *P* |  | N | β (95% CI) ^†^ | *P* |
| **Pro-oxidant genes** | |  | | | | | | | |
| *XDH* | 0 | Increase | 757 | Reference |  |  | 754 | Reference |  |
| (rs4549820) | 1 |  | 36 | 0.05 (-0.31, 0.40) | 0.8 |  | 36 | 0.11 (-0.15, 0.37) | 0.41 |
|  | 2 |  | 0 | - |  |  | 0 | - |  |
| *NOX4* | 0 | Reduce | 351 | Reference |  |  | 351 | Reference |  |
| (rs1083029) | 1 |  | 340 | 0.02 (-0.14, 0.18) | 0.79 |  | 337 | -0.02 (-0.13, 0.10) | 0.79 |
|  | 2 |  | 102 | -0.14 (-0.38, 0.09) | 0.22 |  | 102 | **-0.24 (-0.41, -0.07)** | **0.01** |
| *NFIX* | 0 | Reduce | 775 | Reference |  |  | 772 | Reference |  |
| (rs149677133) | 1 |  | 18 | -0.15 (-0.65, 0.35) | 0.55 |  | 18 | -0.08 (-0.45, 0.29) | 0.67 |
|  | 2 |  | 0 | - |  |  | 0 | - |  |
| *CYP1A1* | 0 | Increase | 337 | Reference |  |  | 337 | Reference |  |
| (rs2470890) | 1 |  | 366 | -0.05 (-0.21, 0.11) | 0.52 |  | 364 | **-0.14 (-0.25, -0.02)** | **0.02** |
|  | 2 |  | 90 | -0.12 (-0.37, 0.13) | 0.34 |  | 89 | -0.05 (-0.24, 0.13) | 0.56 |
|  |  |  |  |  |  |  |  |  |  |
| **Antioxidant genes** | |  | | | | | | | |
| *MAPK10* | 0 | Reduce | 705 | Reference |  |  | 703 | Reference |  |
| (rs80320648) | 1 |  | 84 | 0.06 (-0.18, 0.30) | 0.6 |  | 83 | -0.01 (-0.19, 0.17) | 0.93 |
|  | 2 |  | 4 | 0.46 (-0.58, 1.50) | 0.38 |  | 4 | 0.14 (-0.63, 0.91) | 0.72 |
| *NFKB1* | 0 | Increase | 243 | Reference |  |  | 243 | Reference |  |
| (rs28573147) | 1 |  | 389 | -0.09 (-0.26, 0.08) | 0.29 |  | 388 | -0.02 (-0.15, 0.10) | 0.73 |
|  | 2 |  | 161 | -0.04 (-0.25, 0.17) | 0.72 |  | 159 | 0.01 (-0.15, 0.16) | 0.95 |
| *SP1* | 0 | Increase | 557 | Reference |  |  | 554 | Reference |  |
| (rs35437931) | 1 |  | 218 | -0.09 (-0.25, 0.08) | 0.3 |  | 218 | -0.02 (-0.14, 0.11) | 0.81 |
|  | 2 |  | 18 | 0.12 (-0.38, 0.61) | 0.64 |  | 18 | 0.09 (-0.28, 0.46) | 0.63 |
| *FOS* | 0 | Increase | 336 | Reference |  |  | 333 | Reference |  |
| (rs79713290) | 1 |  | 373 | 0.05 (-0.11, 0.21) | 0.53 |  | 373 | -0.06 (-0.17, 0.06) | 0.34 |
|  | 2 |  | 84 | 0.06 (-0.19, 0.32) | 0.63 |  | 84 | 0.02 (-0.16, 0.21) | 0.8 |
| *CAT* | 0 | Increase | 352 | Reference |  |  | 350 | Reference |  |
| (rs12793666) | 1 |  | 339 | 0.04 (-0.12, 0.20) | 0.62 |  | 338 | -0.02 (-0.13, 0.10) | 0.78 |
|  | 2 |  | 102 | -0.11 (-0.34, 0.12) | 0.35 |  | 102 | -0.05 (-0.22, 0.13) | 0.59 |
| *GPX1* | 0 | Reduce | 223 | Reference |  |  | 221 | Reference |  |
| (rs17650792) | 1 |  | 393 | **0.19 (0.01, 0.36)** | **0.03** |  | 393 | 0.07 (-0.06, 0.20) | 0.3 |
|  | 2 |  | 177 | **0.21 (0.01, 0.42)** | **0.04** |  | 176 | 0.09 (-0.07, 0.25) | 0.26 |
| *SOD1* | 0 | Increase | 627 | Reference |  |  | 624 | Reference |  |
| (rs4998557) | 1 |  | 155 | 0 .00 (-0.18, 0.19) | 0.97 |  | 155 | -0.08 (-0.22, 0.06) | 0.26 |
|  | 2 |  | 11 | -0.14 (-0.77, 0.49) | 0.67 |  | 11 | -0.04 (-0.51, 0.43) | 0.87 |
| *SOD2* | 0 | Increase | 390 | Reference |  |  | 388 | Reference |  |
| (rs5746105) | 1 |  | 333 | 0.04 (-0.11, 0.20) | 0.58 |  | 332 | 0.04 (-0.07, 0.16) | 0.47 |
|  | 2 |  | 70 | 0.10 (-0.17, 0.37) | 0.47 |  | 70 | 0.14 (-0.06, 0.34) | 0.17 |

^*^Pro- and antioxidant genes from the human oxidative-stress response pathway and corresponding SNPs linked to the activity of these genes in brain and other tissue types. SNP rs1083029 (minor allele), which associates negatively with maternal 8-OHdG, reduces the expression of its pro-oxidant target gene *NOX4.* SNP rs17650792, which associates positively with maternal 8-OHGua, reduces expression of its antioxidant target GPX1. ^†^Minimally adjusted for child’s sex and gestational age at urine collection. *8-OHdG 8-hydroxydeoxyguaosine; 8-OHGua 8-hydroxyguanosine; OS oxidative stress; SNPs single-nucleotide polymorphisms.*
